# Fatigue Behaviour of High-Performance Green Epoxy Biocomposite Laminates Reinforced by Optimized Long Sisal Fibers

**DOI:** 10.3390/polym16182630

**Published:** 2024-09-18

**Authors:** B. Zuccarello, C. Militello, F. Bongiorno

**Affiliations:** Department of Engineering, University of Palermo, Viale delle Scienze, 90128 Palermo, Italy; carmelo.militello01@unipa.it (C.M.); francesco.bongiorno01@unipa.it (F.B.)

**Keywords:** biocomposites, natural fiber, sisal fiber, fatigue, life prediction, S-N curve

## Abstract

In recent decades, in order to replace traditional synthetic polymer composites, engineering research has focused on the development of new alternatives such as green biocomposites constituted by an eco-sustainable matrix reinforced by natural fibers. Such innovative biocomposites are divided into two different typologies: random short fiber biocomposites characterized by low mechanical strength, used for non-structural applications such as covering panels, etc., and high-performance biocomposites reinforced by long fibers that can be used for semi-structural and structural applications by replacing traditional materials such as metal (carbon steel and aluminum) or synthetic composites such as fiberglass. The present research work focuses on the high-performance biocomposites reinforced by optimized sisal fibers. In detail, in order to contribute to the extension of their application under fatigue loading, a systematic experimental fatigue test campaign has been accomplished by considering four different lay-up configurations (unidirectional, cross-ply, angle-ply and quasi-isotropic) with volume fraction *V_f_* = 70%. The results analysis found that such laminates exhibit good fatigue performance, with fatigue ratios close to 0.5 for unidirectional and angle-ply (±7.5°) laminates. However, by passing from isotropic to unidirectional lay-up, the fatigue strength increases significantly by about four times; higher increases are revealed in terms of fatigue life. In terms of damage, it has been observed that, thanks to the high quality of the proposed laminates, in any case, the fatigue failure involves the fiber failure, although secondary debonding and delamination can occur, especially in orthotropic and cross-ply lay-up. The comparison with classical synthetic composites and other similar biocomposite has shown that in terms of fatigue ratio, the examined biocomposites exhibit performance comparable with the biocomposites reinforced by the more expensive flax and with common fiberglass. Finally, appropriate models, that can be advantageously used at the design stage, have also been proposed to predict the fatigue behavior of the laminates analyzed.

## 1. Introduction

The vital issue of the continued increase in environmental pollution due to industrial production has led to the emergence of eco-design and the circular economy principle, where the life cycle of a generic industrial product is extended to its disposal and reuse. Regarding the production of new structural materials characterized by low environmental impact [1,2,3,4,5,6,7,8,9,10,11,12,13,14,15,16,17,18], the scientific community has recently looked for an alternative to polymeric composite materials reinforced by synthetic fibers, now widely used in various applications in both industrial and construction sectors. As an example, interesting applications of hybrid pultruded composites under fatigue loading, have been developed in [19] by considering rods for bridge engineering, exhibiting a high fatigue ratio; also, efficient bending processes of novel glass fiber-reinforced polypropylene bending bars have been developed in [20] to improve the mechanical performance of composite bars to be used to replace classical steel bars in generical civil applications or in applications governed by high marine corrosion due to saltwater and chlorides [21]. The so-called high performance biocomposites [22,23,24,25,26,27,28,29,30,31], consisting of low environmental impact matrices reinforced by long natural fibers (renewable materials), are an attractive green alternative to traditional materials, thanks to their low density and high mechanical-specific properties that are in general comparable or superior to those of common metals such as steel and aluminum, or common composites such as fiberglass [1,2,3].

A notable research activity is reported in the literature regarding the development and the mechanical characterization of polymer composites reinforced by natural fibers such as flax, cotton, kenaf, sisal, etc. Generally, the higher mechanical properties have been obtained by using vegetable fibers with higher cellulose content and microfibrils more aligned in the direction of the fiber, as is the case of flax, hemp, kenaf, sisal, jute and ramie. However, the properties of a single fiber commonly depend on several intrinsic factors, such as shape, size, orientation and thickness of the cell walls [4]. The majority of studies reported in the literature mainly concern the development of proper production techniques for these innovative materials and their mechanical characterization, fundamentally by considering only static loading service conditions. Although fatigue service conditions are the most common, especially for structural materials, only very few works have investigated the behavior of biocomposites reinforced by plant fibers under fatigue and/or dynamic service loading [6,7,8,9,10,11,12]. In more detail, such studies have shown that the fatigue strength increases with both the volume percentage of long fibers and the fiber-matrix adhesion. Furthermore, the fatigue behavior of these materials is characterized by good fatigue ratios, generally close to 0.5, essentially related to the quality of the biocomposites (void fraction, etc.) as well as to the environmental parameters (humidity, etc.) that can influence the fiber-matrix adhesion.

Sufficient data are available from multiple sources dealing with the fatigue life of epoxy composites reinforced by flax fibers to enable predictive analysis of several commonly used long fiber lay-up and random short-fiber composites [5].

Also, Liang et al. in [6,7] carried out experimental investigations on flax/epoxy resin samples under tension–tension fatigue loading by varying the stacking sequence and compared the results with the similar epoxy glass-fiber composites. The results showed an excellent adaptation to the Wöhler linear model and, in terms of fatigue strength, values comparable with those of glass-fiber composites at stress levels below 64 MPa. In addition, thanks to the lower specific weight of flax, the specific fatigue strength comparison reveals that the flax fiber composites have better fatigue strength.

In [8], the fatigue damage mechanisms of unidirectional flax composites have been analyzed, and crack propagation has been monitored through infrared thermography. 

In [9], Bensadoun et al. have highlighted how the flax composites with architectures that lead to higher stiffness and strength (accurate alignment of fibers to the load direction) also show an increased fatigue life, delayed damage initiation and a reduced damage propagation rate.

In [10], Gassan demonstrated that fiber-matrix adhesion significantly affects the fatigue behavior of polyester composites reinforced by flax and jute fibers.

Between the works focused on hemp fiber composites [11,12,13,14], in [11] the authors have been the first to fatigue characterize epoxy composite materials reinforced by hemp fiber fabrics, thoroughly analyzing the damage mechanisms.

At the same time, Yuanjian and Isaac [12] have studied the fatigue behavior of a hemp/polyester composite and compared it with a glass/polyester composite with comparable results; despite having a lower absolute fatigue strength, hemp fiber composites have shown higher fatigue ratios. 

Towo and Ansell [15] have characterized fatigue thermosetting composites (polyester and epoxy) reinforced by untreated and NaOH-treated sisal fiber bundles. The results of fatigue tests have shown improvement in the fatigue life of composites following alkali treatment of sisal fiber bundles.

In [16,17], it has been found that the stiffness of vegetable fiber composites (jute, flax and hemp) decreased substantially (up to 50%) at low cycles applied, stabilizing later. However, systematic fatigue tests carried out also on glass fiber composites have shown that the stiffness variation of plant fiber composites is less than that of glass fiber composites.

Recently, in [18], through the hybridization of flax biocomposites with other synthetic fibers (glass, carbon and kevlar), the authors have demonstrated that hybridization opens new possibilities of controlling fatigue and fracture properties of composites. Successive studies are necessary to accurately describe such interesting effects.

In the present work, in order to characterize the actual fatigue behavior of an interesting class of high-performance biocomposites (static tensile strength up to about 500 MPa) made by a “green epoxy” matrix reinforced with optimized long sisal fibers, a systematic campaign of experimental fatigue tests (tension–tension with stress ratio R = 0.1) was carried out by considering four different lay-up configurations (unidirectional, cross-ply, angle-ply and quasi-isotropic) commonly used in practical industrial and civil structural applications. As briefly mentioned above, such high-performance biocomposites have already been widely characterized by the same authors by considering different static loading conditions (tensile, compressive, shear, etc.), low velocity impact, fracture propagation and severe environmental conditions (aging tests). In detail, sisal fibers have been selected because among the various natural fibers (flax, hemp, juta, etc.), they are characterized by several important advantages such as high availability in world trade (about 4.5 million tons of agave fibers is produced worldwide every year), short renewal time, possibility of exploiting marginal lands (it is in practice a weed that does not subtract fertile terrains from agricultural crops), good strength and stiffness, low cost (about 0.3 €/kg), good adhesion with many polymer matrixes, the lowest specific cost in terms of stiffness (about 25 €/GPa.ton), a fibrillar structure that makes it the natural fiber with the highest toughness, comparable with that of glass fibers.

## 2. Biocomposite Materials 

As mentioned above, the biocomposite laminates considered in the present work are composed of a green epoxy matrix reinforced with optimized sisal fibers, whose characteristics are reported in detail in the following sections. 

### 2.1. Green Epoxy Matrix

The matrix used for the manufacturing of the biocomposite laminates considered in the present work is a green epoxy resin (partial biobased epoxy), produced by American Entropy Resin Inc., called SUPERSAP CLR with SuperSap INH Hardener (San Antonio, CA, USA). Such an epoxy resin is produced by using an ecofriendly manufacturing process through green chemistry, sustainable raw materials and efficient manufacturing, conserving energy, minimizing harmful byproducts and reducing greenhouse gas emissions of resins and hardeners. Using Life Cycle Assessment (LCA), the producer has demonstrated how SUPERSAP CLR significantly reduces the environmental impact of the products. As shown in previous studies by the same authors [22,25,26,29], this resin has good mechanical characteristics and good adhesion with the optimized sisal fibers, as demonstrated in [24,27]. This matrix exhibits an almost linear elastic behavior, with the following main characteristics: density *ρ_m_* = 1.05 g/cm^3^, tensile strength *σ_m,R_* = 50 MPa, Young’s modulus *E_m_* = 2.5 GPa and tensile stress strain *ε_m,R_* = 2.5%.

### 2.2. Natural Reinforcement

The sisal fibers used in the manufacturing of the high-performance biocomposite considered in the present work are extracted from the middle third of mature leaves of agave sisalana plants. As it is well known, agave sisalana is a plant belonging to the family of Agavaceae that has elongated pulpy leaves, each reinforced by about a thousand straight fibers arranged longitudinally and concentrated on the perimeter (structural fibers) to form an actual sandwich structure.

**Figure 1:**
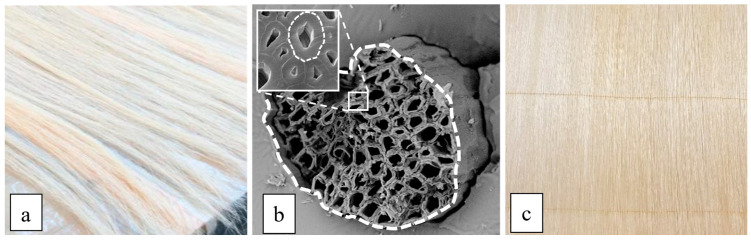
(**a**) Sisal fibers, (**b**) SEM micrographs of the technical fiber cross-section and (**c**) unidirectional sisal-stitched fabrics.

Similar 800–1000 mm long sisal fibers are supplied by Mellau-Teppich Lotteraner, Wüstner GmbH & Co KG (Austria) and obtained from South American plantations. Sisal fibers have a characteristic yellow color (see Figure 1a) and have a specific weight of 14.5 kN/m^3^, significantly lower than that of all synthetic fibers (from approximately 17 kN/m^3^ of the lighter aramid fibers to about 27 kN/m^3^ of the less light glass fibers). For the purpose of maintaining a high degree of renewability, the sisal fibers have only been subjected to manual cleaning and successive drying without any surface treatment. 

The technical (structural) sisal fiber typically comprises many sub-fibers with a diameter varying from 6 to 30 μm. It has a typical horseshoe cross-section (see Figure 1b) with a mean apparent diameter of 150–200 μm (monofilament), different from the usual synthetic fiber diameter (10–12 μm) of the “multi-filament” fiber used in traditional polymer matrix composites (PMCs).

Taking into account the potential variability of the mechanical properties of natural fibers with the particular batch considered, the elastic properties of the fibers used in this work have been determined through specific single fiber tensile tests, carried out in accordance with the ASTM D3822 [32] standard by using an INSTRON 3367 universal testing machine. 

For the batch of fibers used in this study, longitudinal tensile strength values averaging about 680 MPa and a Young’s modulus of about 40 GPa have been obtained.

The biocomposite laminates have been manufactured using a unidirectional *stitched* fabric (see Figure 1c) preliminarily made in the laboratory. In detail, high-quality sisal-stitched fabrics have been implemented through a complex manual process of stretching the fibers, accurate alignment and stitching using an automatic sewing machine. Unidirectional sisal fabrics with a specific weight of about 220 g/m^2^ have been obtained.

### 2.3. Manufacturing of the Biocomposites

**Figure 2:**
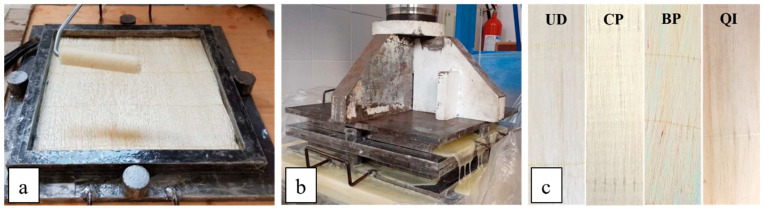
(**a**) Hand lay-up, (**b**) hydraulic press for compression-molding and (**c**) sisal biocomposite laminates obtained by the described optimal manufacturing process.

By using the above-described green epoxy resin and the unidirectional stitched fabrics made in the laboratory, the four different biocomposite laminates have been manufactured through hand lay-up and a subsequent optimized compression-molding process followed by a proper post-cure process. In detail, a suitable mold of 260 mm × 260 mm (see Figure 2a) and a hydraulic press of 100 tons (see Figure 2b). In detail, the initial impregnation of sisal fabrics inside the mold has been carried out in an excess of resin, and the final fiber volume fraction, *V_f_* = 70%, has been obtained by adjusting the final thickness *t_p_* of the biocomposite panel under pressure to the equivalent thickness of the fabrics *t_f_* in accordance with the simple relationship:

(1) $$t_{p}=\frac{t_{f}}{V_{f}}$$

(2) $$t_{f}=\frac{v_{f}}{A_{m}}$$

where and *v_f_* and *A_m_* are the total volume of the fibers and the area of the mold, respectively; as highlighted in Figure 2b, the excess resin leaks out of the mold during the compression-molding process. To obtain high-quality biocomposite laminates with a low void percentage (see Figure 2c), the production of the unidirectional and angle-ply biocomposites with *V_f_* = 70% has been performed by using the optimal gelling time and cure pressure provided in [27]. In more detail, a gelling time *t_g_* = (0.247 + 1.769 *V_f_^5.34^*) *h* and a curing pressure *p_c_* = (42.4 *V_f_^5.45^*) MPa, have been used. Also, as suggested by the matrix supplier, all the biocomposite panels obtained have been subjected to a post-curing process at a controlled temperature of 80 °C for 120 min.

Table 1 shows the actual different stacking sequences used for the four types of analyzed biocomposite laminates. In particular, four different long fiber lay-ups, consisting of 16 oriented laminae with *V_f_* = 70% have been manufactured: unidirectional (UD), cross-ply (CP), braided-ply (BP) and quasi-isotropic (QI).

In detail, the braided-ply lay-up has been considered because the literature [33,34] indicates that similar laminates with reduced angles of misalignment (included between ±5° and ±10°) can exhibit better fatigue performance than unidirectional laminates due to the lower transversal deformation, which corresponds to lower transversal fatigue.

## 3. Experimental Test Methods

### 3.1. Static Tensile Tests

In order to evaluate the tensile strength of each biocomposite laminate (UD, CP, BP, QI) considered, preliminary static tensile tests have been carried out in accordance with the proper standard ASTM D3039 [35]. After bonding proper aluminum tabs in all the tensile specimens (five for each laminate) having dimensions of 25 mm × 250 mm × 3 mm, the tensile tests were performed under displacement control at a standard head displacement rate of 2 mm/min. by using an MTS 810 servo-hydraulic test machine instrumented by an extensometer.

### 3.2. Fatigue Tests

**Figure 3:**
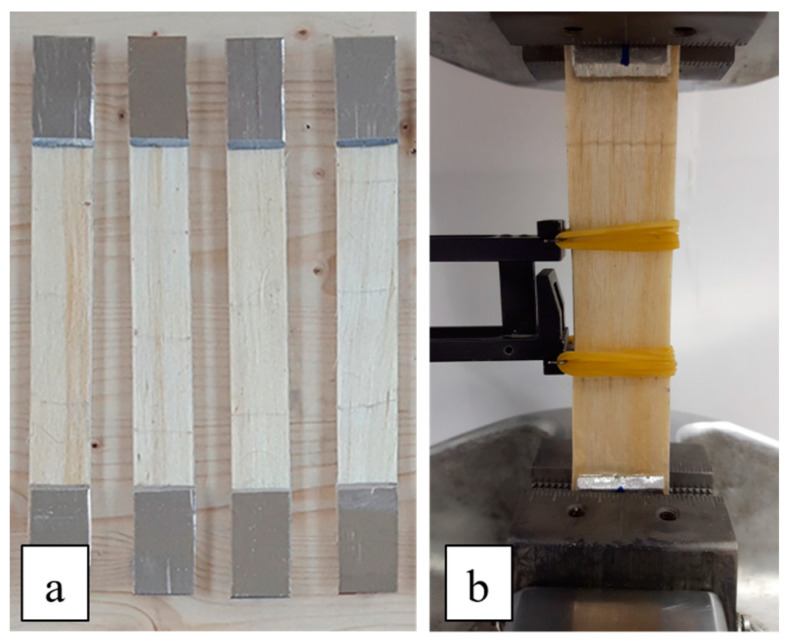
(**a**) rectangular test specimens and (**b**) fatigue specimens under test.

The fatigue tests on biocomposite specimens similar to those described above for the tensile tests have been carried out by using the same servo-hydraulic test machine used for static tests. In more detail, all the rectangular test specimens (see Figure 3a) have been cut by using a circular saw, and the surfaces near the short edges have been properly roughened with sandpaper to avoid possible sliding of the specimens with respect to the grips (see Figure 3b).

Based on static tensile strengths obtained by the preliminary static tensile tests, all the fatigue tests were performed for each laminate by considering four stress levels equal to 80%, 70%, 60% and 50% of tensile strength, respectively; for each laminate, three tests for each stress level have been carried out. The stress level of 40% of tensile strength has been further investigated for cross-ply and quasi-isotropic laminates. A loading frequency of 5 Hz, which ensured the absence of overheating phenomena due to mechanical hysteresis of the material, and a stress ratio of R = 0.1 (tension–tension), have been used. The fatigue tests have been carried out in load control by monitoring the stiffness as the number of cycles varies to evaluate the damage’s evolution and the achievement of the fatigue limit condition, corresponding to the initial stiffness reduction of 30%.

## 4. Analysis of Results and Damage Evaluation

### 4.1. Static Mechanical Properties

**Figure 4:**
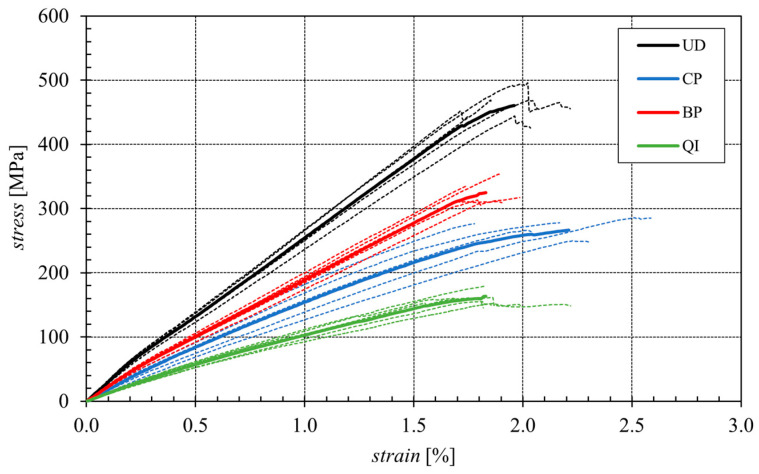
Mean tensile stress curves of the various considered biocomposite laminates.

**Figure 5:**
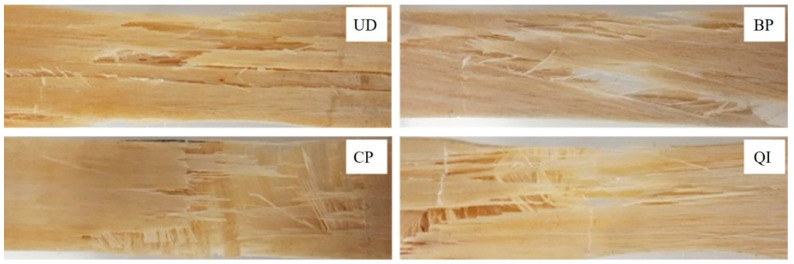
Typical images of specimens fractured by tensile test: UD = unidirectional specimen, BP = braided-ply specimen, CP = cross-ply specimen, QI = quasi-isostropic specimen.

The following Figure 4 shows the average curves obtained from the five tensile tests performed for each type of laminate considered. It is seen how unidirectional and braided-ply biocomposites exhibit linear elastic behavior until failure; in detail, as expected because of the good alignment between fibers and loading axis, unidirectional laminates show the higher tensile strength, between 465 MPa and 500 MPa, with a mean value of 465 MPa, whereas the braided-ply (±7.5°) laminates show tensile strength between 300 and 350 MPa, with a mean value of 326 MPa. It confirms how small angles of fiber-load misalignment significantly reduce the static tensile strength of the laminate due to initiating a primary shear damage mechanism that precedes the fiber failure, as it can be observed in Figure 5. 

The tensile curves relating to the CP and QI laminates highlight instead that such lay-ups exhibit an initial linear elastic phase followed to an elastic-plastic behavior (stiffness decreasing with the load) until failure. Due to the presence of fibers not aligned with the loading axis, the tensile strength values for these types of laminates, equal to about 265 MPa (CP) and 160 MPa (QI), are both significantly lower than those of the unidirectional laminate, with a decrease of about 40% and about 65% for the cross-ply and quasi-isotropic laminates, respectively. 

The same qualitative trend is evident for the value of the longitudinal Young’s modulus, which, for the unidirectional laminate, having all the fibers aligned with the load, assumes values between 25 and 33 MPa (average value equal to approximately 29 GPa), while for the BP, CP and QI laminates, also having fibers not aligned with the applied load, it reduces by around 20%, 40% and 60%, respectively. In terms of failure strain in practice, the same values are observed for all the examined lay-ups, being such a parameter directly related to the final strain failure of the fibers. 

In summary, Table 2 shows the mean ultimate tensile strength and the relative standard deviations of the analyzed biocomposite laminates, as well as the mean longitudinal Young’s modulus and the mean longitudinal failure strain.

Such mechanical performances are in good accordance with those obtained by the authors in previous works [22,26,28], confirming the high quality and repeatability of the manufacturing process used.

The analysis of standard deviations shows that, typically, the scattering of the mechanical properties of these laminates is around 6%, i.e., it is comparable with that of synthetic fiber-reinforced composites and other technical materials. The relatively high scattering values (standard deviations of about 20% or higher) typically found in the single fiber tests are not detected at all because the scattering of the laminate properties does not refer to the single fiber properties but, if anything, to the scattering of the mean value of the thousands of fibers that constitute each specimen. It is easy to demonstrate that the scattering of the mean value of samples constituted by several thousands of elements tends to negligible values so that it is easy to understand that the scattering of the properties of the specimens is not related to the scattering of the single fibers at all but to the amount and distribution of the main defects. Consequently, although the scattering of the mechanical performance of the natural fibers is, as expected, always higher than that of synthetic fibers, the scattering of the materials reinforced by natural fibers is not higher than that of the materials reinforced by synthetic fibers because in both cases the actual scattering is related to the distribution of the defects and not to the scattering of the single fiber properties.

Figure 5 shows the damage observed experimentally at the end of the static tensile tests for each of the four laminates analyzed.

From Figure 5, it is seen how the unidirectional biocomposite (UD) tensile failure is characterized by the longitudinal failure of the fibers without premature appreciable debonding or fiber pull-out phenomena. It is also seen how the transversal propagation of the failure surface is accompanied by secondary debonding phenomena caused by the propagation of the primary transversal cracks along the fiber-matrix interface.

The tensile failure of the BP specimens is manifested as the result of shear matrix failure between the ±7.5° oriented fibers and subsequent fiber failure with evident phenomena of fiber-bridging.

Considering the damaged zone of the CP and QI biocomposite laminates shows that the damage processes occur progressively with preliminary failure of the transverse laminae (90°) and then of the laminae oriented at 45° (in the case of QI lay-up), with appreciable bridging phenomena, followed by the laminae failure oriented with the load (0°) due to longitudinal failure of the fibers.

As expected, instead, the mechanism of failure of the CP laminates is similar to that of the UD laminates, but now it is limited to the longitudinal laminae and follows the preliminary transversal failure of the transversal laminae. Finally, the damage mechanisms of the QI laminate are, in practice, the superposition of the damage mechanisms observed into CP and BP laminates, i.e., the transversal failure of the transversal laminae, followed by the damage mechanism of the BP laminae limited to the ±7.5° oriented laminae, followed again by the final damage of the longitudinal laminae that occurs as in the UD laminates observed above.

### 4.2. Fatigue Life

**Figure 6:**
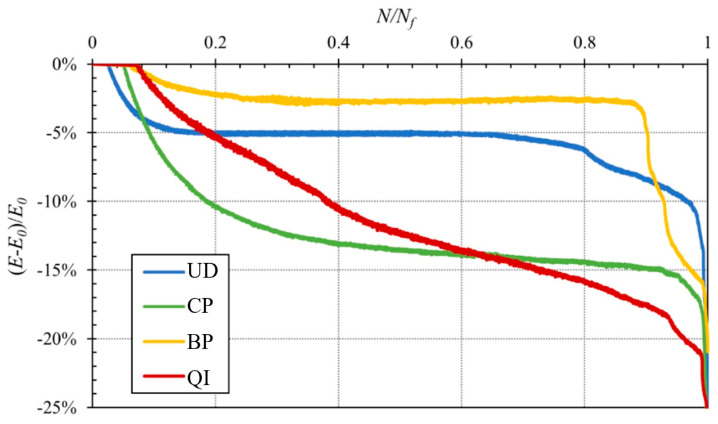
Stiffness degradation as a function of the fraction of the fatigue cycles (*N*/*N_f_*) for a stress level equal to 60% of the static longitudinal tensile strength *σ_L,R_*.

According to the classical literature on composites [36,37], the application of fatigue loading to a generic fiber-reinforced material leads to progressive damage, initially due to microcracking of the transversal laminae, followed by the fatigue damage of the interface and then of the fibers. To these damage phenomena, a progressive reduction in the laminate stiffness occurs. For this reason, for most synthetic fiber-reinforced composite materials, a reliable parameter to evaluate the current health under fatigue load is the so-called percentage damage parameter *D*, defined by the relative variation vof the Young’s modulus, i.e.,

(3) $$D\left(N\right)=100\;\frac{E_{0}-E(N)}{E_{0}}$$

where *E_0_* is the initial longitudinal Young’s modulus, whereas *E*(*N*) is the same Young’s modulus after *N* fatigue cycles. The following Figure 6 shows such damage parameter vs. the fraction of fatigue cycles defined by the ratio between the current fatigue cycles *N* and the final fatigue cycles *N_f_*, for each of the four laminates considered, for a maximum stress level equal to 60% of the static tensile strength. 

From Figure 6, it can be observed how for the UD and BP laminates (fibers almost aligned with the applied load), the damage has an increasing trend up to approximately 15% of the fatigue life, with modest damage values, always less than about 5%, followed by a significant plateau of constant damage; the best performance is exhibited by the BP laminates, with constant damage up to about 85% of the fatigue life (the damage increases instead from about 65% of the fatigue life for UD laminates). For both laminates, a rapid damage evolution occurs in the last 15% of the fatigue life. In other words, for these laminates characterized by a sufficient alignment between load and fibers, the initial degradation corresponds to the damage of the weaker fibers, resulting in a proportional decrease in stiffness. It follows a relatively long period of fatigue defect nucleation inside the fibers with constant stiffness, followed by a final period characterized by the fiber damage with a rapid decrease in the stiffness, i.e., with a significant acceleration of the laminate damage prior to failure. 

In the case of the BP laminate, the reduction in stiffness is quite negligible (about 2%) thanks to the appreciably bridging phenomena produced by the misaligned fibers that limit the initial fiber damage. Such effects also lead also to a longer plateau with negligible fatigue damage. 

The fatigue behavior is instead quite different for cross-ply (CP) and quasi-isotropic (QI) laminates, also being influenced by the non-aligned laminae (at 90° and ±45°). 

In more detail, for the CP laminates, the damage curves have an initial period (up to about 40% of the fatigue life) characterized by a decreasing slope that corresponds to the fatigue damage of the transversal laminae with a stiffness decreasing of about 15%; it follows a second period (up to about 90% of the fatigue life) with almost constant damage (plateau) that corresponds to the nucleation of fatigue defects on the fibers of the aligned laminae, nucleation that leads to the final period characterized by the fatigue fiber failure, which corresponds to a curve having a rapid increasing slope, until failure. 

Finally, the BP laminates exhibit the more complex stiffness degradation curve, being constituted by a first little period (up to 20% of the fatigue life) characterized by an increasing slope with limited damage (up to about 5%) related to the transversal laminae, followed by a relatively long period corresponding to the progressive damage of the ±45% laminae (characterized by a quite constant slope) that, along with the contemporary nucleation of the defects on the fibers of the longitudinal laminae, leads to the final period (last 10% of the fatigue life) characterized by the typical rapidly increasing slope up to the laminate failure.

It is important to highlight how the fatigue life of the analyzed laminates corresponds in practice to the evolution of the stiffness degradation until about 25%; such a value is not very different from that observed for synthetic fiber composites (about 30%). 

**Figure 7:**
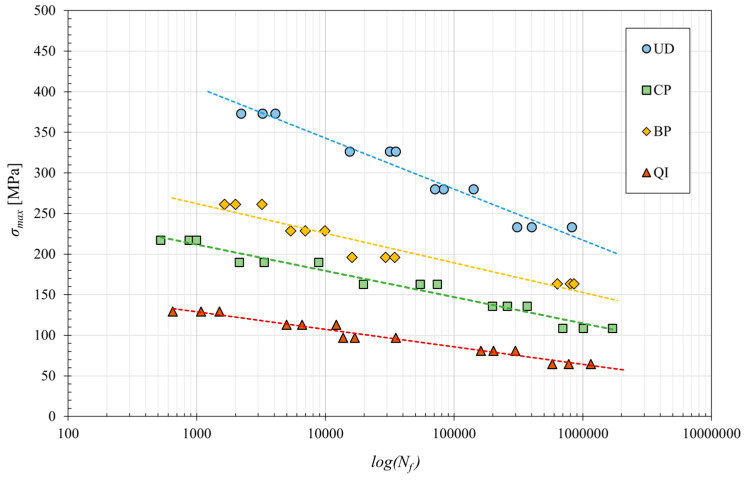
S-N curves of the various analyzed biocomposite laminates.

From a quantitative comparative examination of the curves in Figure 6, it is possible to conclude that the best fatigue performances are shown by the BP laminate. For this lay-up, about 85% of the fatigue life passes with negligible damage, not exceeding 2.5%. However, because the static strength of the BP laminates is inferior to that of the UD laminates, as it can be in the following fatigue curves (Figure 7), the absolute fatigue performance of the UD is higher than that of the BP. 

Figure 7 shows the classical fatigue curves for the four laminates considered, obtained by linear interpolating the experimental data reported in a classic S-N lifetime diagram.

From the analysis of the fatigue curves (see Figure 7), it can be immediately observed that they can be accurately approximated by the semi-logarithmic linear model represented by the well-known equation called modified Wohler law [33,34]:

(4) $$\sigma_{max}=a+b\;log(N_{f})$$

where *a* and *b* are two constants which, assuming that the material has a fatigue limit *σ_F_* at 10^6^ cycles, would theoretically take the static tensile strength value *σ_L,R_* and (*σ_F_* − *σ_L,R_*)/6, respectively. Unfortunately, because the behavior at low fatigue cycles is different from that at high fatigue cycles, the two constants *a* and *b* have always been accurately determined by considering the test results for high fatigue cycles, i.e., for *N_f_* > 10^3^. The values thus defined for the four biocomposites considered have been reported in Table 3.

From Figure 7, it is seen that for fatigue at the high number of cycles (>10^3^ cycles), the best absolute performances are exhibited by the UD laminates, which have a fatigue strength at 10^6^ cycles of about 220 MPa; it follows the BP laminates with a lower fatigue strength of more than −30% (about 150 MPa), then the CP laminates with a fatigue strength of about 115 MPa (about −50% compared to laminate UD); finally, the QI laminate with a fatigue strength of about 65 MPa (−70% compared to UD laminate) is that with the lower fatigue performance. 

Table 3 shows the values of static strength (*σ_L,R_*), fatigue limit (*σ_F_*) at 10^6^ cycles, characteristic fatigue ratio (*σ_F_*/*σ_L,R_*) and constants *a* and *b* for the different laminates.

Notably, the highest fatigue ratios, approximately 0.5, close to the commonly observed values for technical metals and GFRP, were exhibited by laminates with fibers aligned with the load (UD and BP). Also, relatively good fatigue performances are exhibited by the CP and QI laminates, with values of the fatigue ratio close to or slightly higher than 0.4. However, all these values are very interesting for structural and semi-structural applications: the unidirectional lay-up allows the user to obtain biocomposites for one-dimensional structures (beams, etc.) with a fatigue strength comparable to that of standard structural steels (220 MPa). The UD lay-up can be advantageously replaced by the BP lay-up in the presence of a limited transversal fatigue load, whereas the CP lay-up has to be used for biaxial fatigue loading, and the QI or equivalent random lay-up with discontinuous fibers (MAT) has to be used for the generic application of reinforced plastics under variable loading; the fatigue strength of QI (65 MPa) is, in fact, more than the value of the static strength of the simple matrix and about five times superior to its fatigue strength (about 20% of the static strength). 

To allow a comparison of the fatigue behavior of the different laminates, it is helpful to verify the trend in the non-dimensional fatigue curves corresponding to the model represented by the following equation:

(5) $$\sigma_{max}/\sigma_{L,R}=a’+b’\;log(N_{f})$$

**Figure 8:**
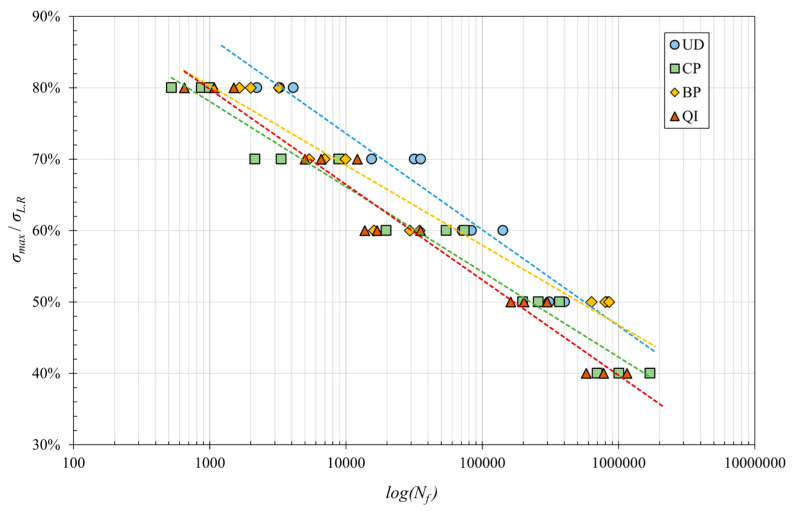
Non-dimensional fatigue curves for the different laminates considered.

The following Figure 8 shows the trend in these curves for the four different laminates considered in this study.

Figure 8 allows the evaluation of a given material’s fatigue performance concerning its static performance. It has been possible to observe how, in a low number of cycles (around 10^3^ cycles and up to 10^4^ cycles), CP laminates perform less than QI laminates, which in turn are less fulfilling than BP and UD. It has also been significant to observe that for the high number of cycles (around 10^6^), the relative performance of BP (±7.5°) laminates exceeds that of unidirectional (0°). The interpolation of the experimental data for the four different laminates with Equation (3) gives the values of the coefficients *a*’ and *b*’ reported in Table 3.

Since, in general, Equations (4) and (5) do not describe fatigue behavior at low fatigue number of cycles (*N_f_* < 10^3^), as it is also the case for biocomposites examined, that is, they adapt well to the experimental data for high fatigue cycles but do not converge to static strength, several complete models able to represent both the low and the high fatigue behavior have been proposed in the literature. However, in the literature [38], the following model of the S-N curve of Kim and Zhang [38] has been evaluated as the most suitable for the fatigue life of composite materials:

(6) $$\sigma_{max}=\sigma_{L,R}\;{\left(\frac{\alpha \left(\beta -1\right)\left(N_{f}-N_{0}\right)}{{\sigma_{L,R}}^{-\beta }}+1\right)}^{1/\left(1-\beta \right)}$$

where *α*, *β* are the model fitting parameters, and the term *N*_0_ is used to adjust the initial number of cycles for the failure point of the first cycle.

However, in [11], Vasconcellos et al. pointed out that the following model proposed in [39] by D’Amore et al. for synthetic fiber composites can also be used for the complete prediction of fatigue life (low cycles and high cycles) of biocomposites reinforced with hemp fibers:

(7) $$\sigma_{max}=\frac{\sigma_{L,R}}{\alpha ’\left(N_{f}^{\beta^{’}}-1\right)\left(1-R\right)+1}$$

where *α* and *β* are the model fitting parameters and *R* is the stress ratio.

The following Table 4 shows the values of the constants describing the complete fatigue behavior of the biocomposites considered according to the models previously reported.

**Figure 9:**
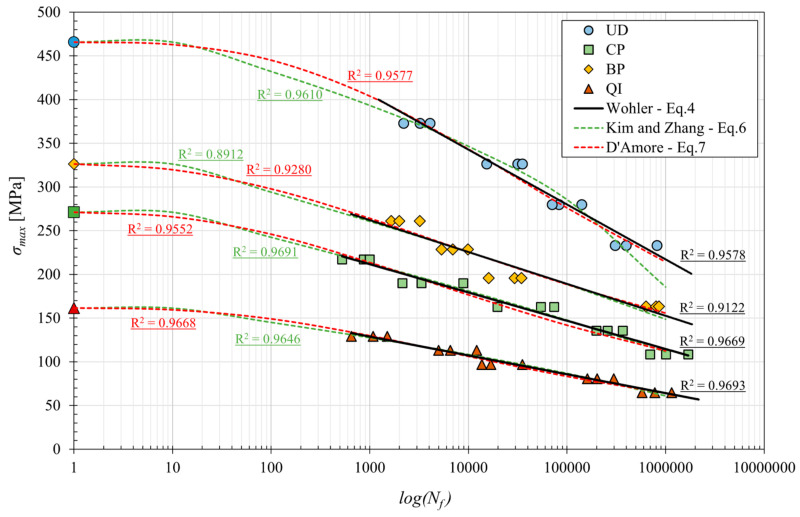
S-N curves of the various analyzed biocomposite laminates, obtained with the different models.

In order to compare the different models of the S-N curves reported, Figure 9 shows the relative curves obtained with the model proposed by Kim and Zhang with Equation (6) and that of D’Amore et al. with Equation (7) for each lay-up, superimposed on the classic model of Equation (4) and the respective experimental points.

From Figure 9, it is possible to notice how the model of Kim and Zhang and the model of D’Amore et al. both provide a good approximation of the fatigue behavior for all examined biocomposites, from the case of static load (*N_f_* = 1) to the conventional fatigue limit at *N_f_* = 10^6^ cycles. In more detail, better correspondence with the experimental data is observed with the model proposed by D’Amore et al. in the case of UD laminates. The difference between the two models, instead, is negligible in the other cases of CP, BP and QI angle-ply laminates. Therefore, it is possible to state that the model proposed by D’Amore et al. can be valid for predicting fatigue behavior for any stress level and for all possible lay-ups of high-performance sisal fiber biocomposites considered.

**Figure 10:**
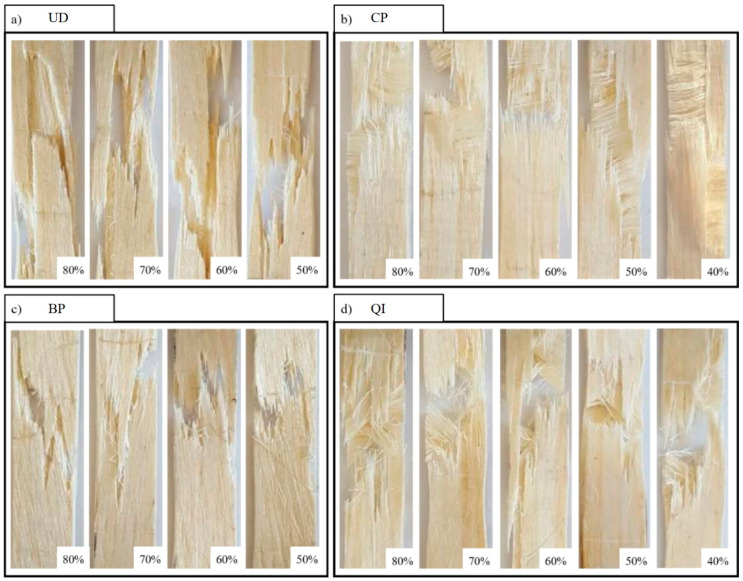
Fatigue-damaged specimens at different percentage stress levels: (**a**) unidirectional, (**b**) cross-ply, (**c**) braided-ply at ±7.5° and (**d**) quasi-isotropic.

Concerning fatigue damage mechanisms, as mentioned above, it is possible to state that the experimental analysis has shown that these are not very different from the mechanisms observed in static loading conditions. For the four lay-ups considered, Figure 10 shows the images of the damaged specimens at the different percentage stress levels applied.

From Figure 10a, it is seen how the fatigue damage mechanism of UD laminates essentially involves the progressive failure of the fibers, most of which occurs in the last part of the fatigue life with unavoidable secondary (due to the obvious crack propagation at the interface of the initial orthogonal matrix crack) debonding phenomena. It is in practice the same damage mechanism previously observed in the presence of static loading; however, the absence of appreciable micro-cracking phenomena, which typically occur in the fatigue of similar materials in the presence of matrix defects (voids, inclusions), essentially confirms the excellent quality of the analyzed biocomposite laminates. Similar damage mechanisms are observed for BP laminates, as shown in Figure 10c, which shows the images of the damaged specimens at different applied maximum stress levels. In detail, it is crucial to observe how BP laminates have more limited fiber-matrix debonding mechanisms compared to UD laminates, the propagation of these phenomena being prevented by the interweaving of inclined fibers (bridging), which generally contrasts the transversal failure mechanisms that can lead to early failure of the laminate. From Figure 10b, it can be observed that for the cross-ply configuration, fatigue damage occurs, as already marked based on the variations of tensile stiffness of the specimens (see Figure 6), for the subsequent failure of the orthogonal laminae and therefore of the longitudinal ones. Also, in the case of QI laminate (see Figure 10d), as already observed based on the variations in tensile stiffness of the relative test specimens (see Figure 6), the fatigue damage is progressive due to subsequent failure of the orthogonal laminae (25% of the total laminae), then of the laminae to ±45° (50% of the total laminae) and finally of the longitudinal ones (last 25%).

## 5. Comparison with Other Natural and Synthetic Fiber Composites

In order to compare the fatigue performance of the analyzed biocomposites analyzed with those of other composite materials reinforced by natural and synthetic fibers, the principal results available in the literature for these materials have been reported in Table 5. In detail, for each considered laminate composite, Table 5 shows the laminate type (fiber/matrix), the lay-up, the fiber volume fraction *V_f, the_* static tensile strength, the fatigue strength limit value at 10^6^ cycles, and the relative characteristic fatigue ratio (*σ_F_/σ_L,R_*).

**Figure 11:**
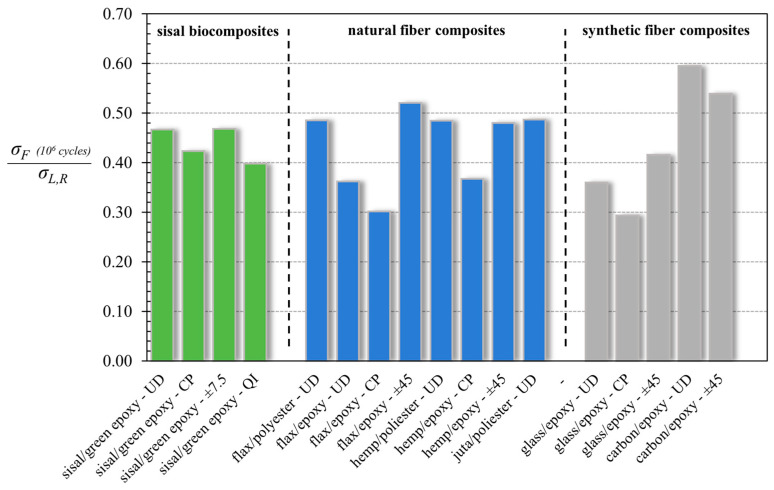
Comparison of the fatigue ratio of the analyzed biocomposites with that of other composites reported in the literature [6,7,11,17,36,40,41].

For a more immediate comparison of the fatigue performance of the composites considered in Table 5, in Figure 11, the relative values of the fatigue ratio (*σ_F_*/*σ_L,R_*) have also been reported through a bar graph.

Obviously, the comparison refers to composite materials having different fiber volume fractions; that is, a parameter that can significantly influence the fatigue performance of a fixed material, which in general is also strictly related to the specific manufacturing method (pultrusion, compression-molding, hand-layup, etc.), as well as to the particular matrix used (thermoplastic, thermosetting), so it is a characteristic of the materials. However, the comparison considers the fatigue ratio, which introduces it to a sort of normalization, being in practice the ratio between fatigue performance and static performance related strictly to *V_f_*. 

First of all, from the analysis of the values reported in Table 5 and in Figure 11, it is possible to observe how the high-performance sisal biocomposites analyzed in the current work (green columns, fatigue ratio between about 0.4 and 0.5) exhibit fatigue ratios comparable to those of the natural fiber biocomposites (flax, hemp and juta) reported in the literature (blue columns, fatigue ratios between 0.3 and 0.52). With reference to the synthetic composites instead, it is possible to observe how the analyzed biocomposites have fatigue ratios higher than those of fiberglass composites (fatigue ratio in the range of 0.3–0.4), whereas, as expected due to the high stiffness of carbon fibers, higher fatigue ratios are exhibited by high-cost carbon fiber composites.

**Figure 12:**
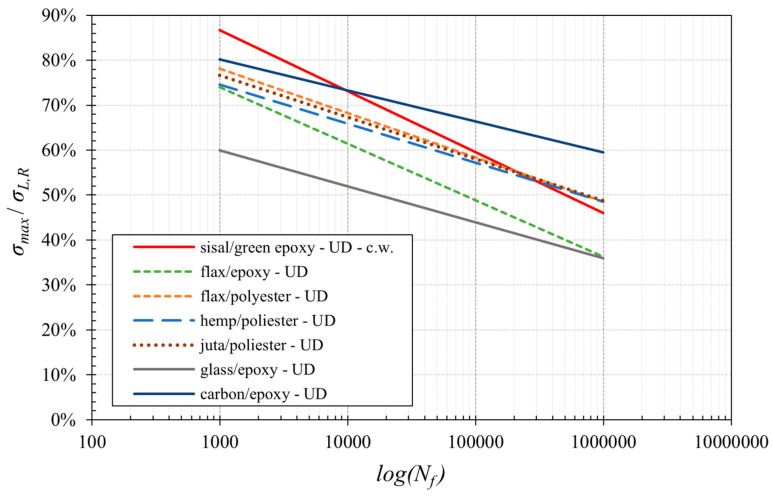
Comparison of the normalized S–N curve of the analyzed unidirectional biocomposite with other UD composites reported in the literature [6,7,17,40,41].

For a more detailed comparison, in Figure 12, the normalized S-N curves of the best performing composites, i.e., of the analyzed UD biocomposites and the unidirectional composites considered in Table 5, have been reported.

From Figure 12, it is clear how the fatigue behavior of the UD sisal fiber biocomposite at low numbers of cycles performs better than all the other considered composites, included carbon composites characterized by their high cost and environmental impact. This very interesting result, that is maintained up to about 10^4^ fatigue cycles, is due to the sub-fibrillar structure of the sisal fiber (very similar to that of aramidic fibers) that leads to high failure energy and high fracture strength. Unfortunately, the relatively reduced stiffness of the analyzed biocomposites leads to the loss of such an advantage for very high fatigue cycles (more than about 3 10^5^ fatigue cycles) with respect to the high-quality, more stiff natural fibers such as flax, etc.

It is interesting to note that at high fatigue cycle numbers, as with other natural fiber biocomposites, the biocomposites developed in the present work exhibit fatigue ratios that are significantly higher (about +27%) than those of traditional fiberglass-reinforced composites; such results confirm how the proposed high-performance biocomposites can be advantageously used to replace common fiberglass not only in terms of static loading [27], impact [26] and fracture [25], but also in terms of fatigue loading. 

## 6. Conclusions

Taking into account that the four different high performance (tensile strength included between 161.5 and 465.8 MPa) lay-ups considered in the present work, i.e., unidirectional ([0]_16_), cross-ply([(0/90)_4_]_s_), braided-ply([(±7.5)_4_]_s_) and quasi-isotropic ([(0/±45/90)_2_]_s_), are able to represent practically all the different stacking sequences that are potentially usable in the ordinary industrial design of structural components subject to static and/or fatigue loading, it is possible to state that:

- The laminates exhibit a good fatigue performance, with fatigue ratios close to 0.5 for unidirectional and angle-ply (±7.5°) laminates and close to 0.4 for cross-ply and quasi-isotropic laminates. Interestingly, the absolute fatigue strength values at 10^6^ cycles are equal to about 220 MPa, 150 MPa, 115 MPa and 65 MPa, respectively, for unidirectional, braided-ply, cross-ply and quasi-isotropic lay-up.
- Such fatigue performances are comparable to those of ordinary structural steels and are better than those of different aluminum alloys; consequently, the UD laminates can be used to advantageously replace steel and aluminum in structural applications related to components subject to both static and fatigue loading.
- Unlike described in the literature in relation to synthetic fiber composites, although the braided-ply layup exhibits the best relative fatigue behavior (negligible damage until 85% of the fatigue life), it does not provide the best absolute fatigue strength due to the significant relative reduction in the static strength.
- Interestingly, the fatigue strength of 150 MPa of cross-ply laminate indicates that such lay-up can be widely exploited in the design of structural and semi-structural mechanical components subject to biaxial fatigue loading, as does the fatigue strength of 65 MPa of the quasi-isotropic laminate, which is about 4–5 times the fatigue strength of the matrix alone, indicating that such laminates (or equivalent random discontinuous fiber configurations, the so-called MAT) can be advantageously used to replace plastics in the presence of generic fatigue loading.
- Appropriate models for predicting fatigue behavior at high and low numbers of cycles have been proposed.
- The comparison with both natural fiber and synthetic fiber composites reported in the literature has highlighted that at “low cycles number” fatigue the analyzed biocomposites exhibit better performance than all the comparable composites reported in the literature, also including high-cost and high-environmental impact carbon composites. Such an advantage is preserved up to about 3 10^5^ cycles for all other composites, and it is lost for higher fatigue cycles with respect to high-cost and high-stiffness natural fiber biocomposites.
- The fatigue performances of the analyzed biocomposites are always superior to those of the common fiberglass; such a result confirms previous results that have already been reported in the literature by the same authors, which show in detail that the proposed high-performance biocomposites can be advantageously used to replace common fiberglass in order to increase the use of green materials.

To further improve the fatigue strength, several successive studies could be implemented by considering: (a) the introduction of green filler (biochar, etc.) to further improve further fiber–matrix adhesion, (b) surface hybridization to reduce the environmental effects, especially for outside applications and (c) the use of thermoplastic matrixes to increase recyclability. 

## Figures and Tables

**Table 1:** Lay-ups of the four implemented biocomposites.

Laminate	Acronimus	Lay-up
Unidirectional	UD	[0]_16_
Cross-ply	CP	[(0/90)_4_]_s_
Braided-ply	BP	[(±7.5)_4_]_s_
Quasi-isotropic	QI	[(0/±45/90)_2_]_s_

**Table 2:** Average mechanical properties of the various considered biocomposite laminates.

	Laminate			
	UD	BP	CP	QI
Ultimate Tensile Strength *σ_L,R_* [MPa] SD of the Ultimate Tensile Strength [MPa]	465.8 17.7	326.4 15.9	271.2 25.7	161.5 9.3
Young’s modulus *E_L_* [GPa] SD of the Young’s modulus [GPa]	26.4 1.08	20.1 0.94	16.9 2.41	11.4 0.84
Failure Strain *ε_L,R_* [%] SD of the Failure Strain [%]	1.9 0.11	1.8 0.10	2.2 0.24	1.8 0.06

**Table 3:** Static strength, fatigue limit and fatigue ratios of the analyzed biocomposites.

Lay-up	*σ_L,R_* [MPa]	*σ_F_* [MPa]	Fatigue Ratio	*a* [MPa]	*b* [MPa]	*a’*	*b’*
UD	465.8	217.2	0.47	594.1	−62.8	1.275	−0.134
CP	271.2	114.7	0.42	308.8	−32.3	1.139	−0.119
BP	326.4	152.7	0.47	371.6	−36.5	1.138	−0.112
QI	161.5	64.2	0.4	193.7	−21.6	1.198	−0.133

**Table 4:** Model fitting parameters according to the Kim and Zhang and D’Amore et al. models.

	Kim and Zhang		D’Amore et al.	
Lay-up	*α*	*β*	*α’*	*β’*
UD	2.292	−1.697	0.0067	2.9423
CP	0.7121	−0.343	0.0217	2.3878
BP	0.7448	−0.352	0.0228	2.2210
QI	2.3420	−0.624	0.0149	2.6401

**Table 5:** Comparison of the fatigue performances of the analyzed biocomposites with those of other composites reinforced by natural and synthetic fiber reported in the literature.

Laminate	Lay-up	*V_f_* [%]	*σ_L,R_* [MPa]	*σ_F_* (10^6^ Cycles) [MPa]	Fatigue Ratio	Refs.
*Sisal fiber biocomposites (from present work)*						
sisal/green epoxy	[0]_16_	70	465.8	217.2	0.466	current work
sisal/green epoxy	[0/90]_4S_	70	271.2	114.7	0.423	current work
sisal/green epoxy	[±7.5]_4S_	70	326.4	152.7	0.468	current work
sisal/green epoxy	[0/±45/90]_2S_	70	161.5	64.2	0.398	current work
*Other natural fiber composites (from literature)*						
flax/polyester	[0]_4_	27	263.3	114.6	0.485	[17]
flax/epoxy	[0]_12_	43	318.0	115.2	0.632	[6,7]
flax/epoxy	[0/90]_3S_	43	170.0	51.2	0.301	[6,7]
flax/epoxy	[±45]_3S_	43	79.0	41.1	0.520	[6,7]
hemp/poliester	[0]_4_	36	171.3	83.1	0.485	[17]
hemp/epoxy	[0/90]_7_	36	113.0	41.5	0.367	[11]
hemp/epoxy	[±45]_7_	36	66.0	31.7	0.480	[11]
juta/poliester	[0]_4_	32	175.1	85.3	0.487	[17]
*Synthetic fiber composites (from literature)*						
glass/epoxy	[0]_5_	30	570.0	205.1	0.360	[40]
glass/epoxy	[0/90]_3S_	43	380.0	111.6	0.294	[6,7]
glass/epoxy	[±45]_3S_	43	103.0	42.8	0.415	[6,7]
carbon/epoxy	[0]_12_	64	1934.0	1150.0	0.595	[41]
carbon/epoxy	[±45]_2S_	58	188.7	101.7	0.539	[36]

## Data Availability

Data available on request from the corresponding author (due to privacy reasons).
